# The complement receptor C5aR2 regulates neutrophil activation and function contributing to neutrophil-driven epidermolysis bullosa acquisita

**DOI:** 10.3389/fimmu.2023.1197709

**Published:** 2023-05-19

**Authors:** Daniel L. Seiler, Katja H. Kähler, Marie Kleingarn, Christian D. Sadik, Katja Bieber, Jörg Köhl, Ralf J. Ludwig, Christian M. Karsten

**Affiliations:** ^1^ Institute for Systemic Inflammation Research (ISEF), University of Lübeck, Lübeck, Germany; ^2^ Center for Research on Inflammation of the Skin (CRIS), University of Lübeck, Lübeck, Germany; ^3^ Department of Dermatology, Allergology and Venerology, University Hospital Schleswig-Holstein, Lübeck, Germany; ^4^ Lübeck Institute of Experimental Dermatology (LIED), University of Lübeck, Lübeck, Germany; ^5^ Division of Immunobiology, Cincinnati Children’s Hospital Medical Centre, University of Cincinnati College of Medicine, Cincinnati, OH, United States

**Keywords:** C5aR2, neutrophil, FcgRIIB, EBA, C5a, CD11b, Mac-1, Fcγ receptor (FcγR)

## Abstract

**Introduction:**

The function of the second receptor for the complement cleavage product C5a, C5aR2, is poorly understood and often neglected in the immunological context. Using mice with a global deficiency of *C5aR2*, we have previously reported an important role of this receptor in the pathogenesis of the neutrophil-driven autoimmune disease *epidermolysis bullosa acquisita* (EBA). Based on *in vitro* analyses, we hypothesized that the absence of C5aR2 specifically on neutrophils is the cause of the observed differences. Here, we report the generation of a new mouse line with a LysM-specific deficiency of *C5aR2*.

**Methods:**

LysM-specific deletion of *C5aR2* was achieved by crossing *LysM^cre^* mice with *tdTomato-C5ar2^fl/fl^* mice in which the *tdTomato-C5ar2* gene is flanked by loxP sites. Passive EBA was induced by subcutaneous injection of rabbit anti-mouse collagen type VII IgG. The effects of targeted deletion of *C5ar2* on C5a-induced effector functions of neutrophils were examined in *in vitro* assays.

**Results:**

We confirm the successful deletion of C5aR2 at both the genetic and protein levels in neutrophils. The mice appeared healthy and the expression of C5aR1 in bone marrow and blood neutrophils was not negatively affected by LysM-specific deletion of C5aR2. Using the antibody transfer mouse model of EBA, we found that the absence of *C5aR2* in LysM-positive cells resulted in an overall amelioration of disease progression, similar to what we had previously found in mice with global deficiency of *C5aR2*. Neutrophils lacking C5aR2 showed decreased activation after C5a stimulation and increased expression of the inhibitory Fcγ receptor FcγRIIb.

**Discussion:**

Overall, with the data presented here, we confirm and extend our previous findings and show that C5aR2 in neutrophils regulates their activation and function in response to C5a by potentially affecting the expression of Fcγ receptors and CD11b. Thus, C5aR2 regulates the finely tuned interaction network between immune complexes, Fcγ receptors, CD11b, and C5aR1 that is important for neutrophil recruitment and sustained activation. This underscores the importance of C5aR2 in the pathogenesis of neutrophil-mediated autoimmune diseases.

## 1 Introduction

The potent anaphylatoxin C5a plays a critical role in promoting and sustaining inflammation by recruiting and activating immune cells that express C5a receptors (C5aRs; 1–4). In plasma or tissues, the C-terminal arginine (Arg) residue of C5a is readily cleaved by the proteolytic activity of carboxypeptidases, resulting in C5a^desArg^ (5–7). C5a and to a lesser extent C5a^desArg^ can bind to two receptors, C5aR1 (CD88) and C5aR2 (C5L2, GPR77). Both receptors contain seven transmembrane domains and therefore belong to the group of G protein-coupled receptors (GPCRs). However, despite their classification as GPCRs, only C5aR1, but not C5aR2, can signal through G proteins (8, 9). This difference is mainly attributed to three factors: (I) the replacement of an arginine residue by a leucine residue in the DRY motif, which is critical for Gα-protein coupling, (II) the absence of serine/threonine residues in the third intracellular loop mediating G-protein recognition in C5aR1, and (III) a modification in the NPXXY motif of the seventh transmembrane helix, acting as an important signal transduction sequence in GPCRs (8, 10–15). However, it has been shown that C5aR2 can modulate the recruitment of β-arrestin to C5aR1, and also recruit β-arrestin itself (16–18). It has therefore been suggested that it should be correctly termed an arrestin-coupling receptor (ACR; 9). Interestingly, this study also showed that the site of phosphorylation is dependent on the ligand, the ligand-binding receptor, and the cells expressing these receptors. This underscores an important finding from previous studies that downstream signaling and receptor internalization of C5a receptors and other GPCRs can be precisely controlled by the number and location of phosphorylations (9, 16, 19). Another potential mode of regulation of downstream signaling has been described at the level of β-arrestins themselves, as different binding modes and conformations of β-arrestins upon binding to a GPCR or ACR have been associated with their multifunctionality and functional diversity (9, 20–23). This might partially explain the controversy over the biological and pathological function of C5aR2, with both pro- and anti-inflammatory properties reported. Studies of LPS- (24) and IC-mediated lung injury (25), allergic contact dermatitis (26), and intestinal ischemia-reperfusion injury (27) suggest anti-inflammatory properties of C5aR2. By contrast, studies in sepsis (28), experimental allergic asthma (29), thioglycolate-induced peritonitis and air-pouch inflammation (30), and renal ischemia-reperfusion injury (31, 32), among others, suggest pro-inflammatory functions for C5aR2. Moreover, in a disease model of immune complex (IC)-induced arthritis, C5aR2 has been shown to be required for the transport of C5a into the blood vessel lumen and therefore critically involved in the recruitment and adhesion of circulating leukocytes, a fundamental requirement for tissue inflammation (33, 34). In line with these latter findings, we recently reported a net pro-inflammatory contribution of C5aR2 to the pathogenesis of *epidermolysis bullosa acquisita* (EBA; 35). EBA is a rare autoimmune disease of the skin that belongs to the group of pemphigoid diseases and is characterized by tense blisters and erosions. The autoantibodies in EBA are directed against type VII collagen (COL7) an important structural protein that stabilizes the dermal-epidermal junction zone (36). The ICs formed by the binding of the autoantibodies to their target antigen activate the complement system, mainly through the classical and alternative pathways (36, 37). Activation of the complement system leads to exponential local generation of the anaphylatoxin C5a, which has been identified as critical for EBA pathogenesis by inducing leukocyte recruitment and activation (38, 39). Previously, global C5aR1-deficient mice have been shown to be almost completely protected from disease development in a mouse model that closely resembles the effector phase (39–41). In the same mouse model, we recently showed that global C5aR2 deficiency significantly ameliorated the disease development, although the effect was not as pronounced as with global C5aR1 deficiency (35).

Interestingly, when we examined the activation potential of neutrophils from mice with global deficiency in *C5ar2 in vitro*, we found reduced CD11b up-regulation after C5a stimulation (35). CD11b is a unique α-subunit (α_m_) that non-covalently couples to a common β_2_-subunit (CD18) to form Mac-1 (macrophage-1 antigen, CD11b/CD18, CR3), a member of the CD18 family of integrins found mainly on granulocytes and monocytes/macrophages (42). Using mice deficient in Mac-1, it has been shown that this integrin supports adhesive functions that contribute to leukocyte recruitment and spreading as well as pathogen clearance by phagocytosis and reactive oxygen species (ROS) generation (43, 44). Ligands for Mac-1 include the intracellular adhesion molecule-1 (ICAM-1), a leukocyte adhesion receptor found on endothelial cells, the inactivation product of C3b, iC3b, the matrix molecule heparin, and factors of the coagulation system such as fibrinogen (45). Unlike other members of the CD18 family, such as lymphocyte function-associated antigen 1 (LFA-1), Mac-1 is stored in secretory vesicles in neutrophils that are transported to the cell surface once the cell receives an activating stimulus (46), e.g., through the C5a/C5aR1-axis.

The importance of Mac-1 for neutrophil recruitment to the skin and subsequent disease development has also been demonstrated in the pathogenesis of EBA and bullous pemphigoid (BP), another pemphigoid disease. *Cd18^–/–^* mice exhibited defective recruitment of neutrophils to the skin and were resistant to disease development in the antibody transfer model of EBA (47). Using the same mice as well as specific antibody blockade of CD18, an elegant study showed that IC-mediated neutrophil adhesion to target tissues is mediated by CD18 and creates a closed, protected space in which proteinases and ROS can exert their tissue-damaging effects (48). In a neonatal antibody transfer mouse model of BP, neutrophil infiltration was also significantly reduced in Mac-1-deficient mice or when CD11b or CD18 was targeted by neutralizing antibodies (49). The latter study also found that neutrophil degranulation, particularly release of the proteases neutrophil elastase and matrix metallopeptidase 9 (MMP-9), was impaired in mice lacking Mac-1 (49). These studies illustrate that Mac-1 is not only involved in neutrophil migration in these disease settings, but also affects important effector functions of neutrophils.

Since neutrophils are the major effector cells in EBA that are recruited and activated, releasing ROS and proteases that ultimately lead to tissue damage, based on our *in vitro* activation assays, we previously hypothesized that the effects of global *C5ar2* deficiency observed in the EBA mouse model were due to impaired neutrophil function in the absence of C5aR2. Here, we report the generation of a new mouse line with a myeloid-specific deletion of *C5aR2*. Mice of this strain were then used for the EBA mouse model, the results of which we also report here to further support our previously published data and hypotheses.

## 2 Materials and methods

### 2.1 Mice and study approval

Mice of both sexes of the mouse strains *C57BL/6J* (WT) as well as *C5ar2-tdTomato^fl/fl^* and *LysM^cre^-C5ar2^–/–^*, the latter both on the genetic background *C57BL/6J*, were used for this study. Mice were bred and housed in the animal facility of the University of Lübeck on a 12-hour light-dark cycle. Injections were performed on 8- to 12-week-old animals after anesthesia. All animal experiments were performed according to the guidelines of the German Society for Laboratory Animal Science and the European Health Law of the Federation of Laboratory Animal Science Associations. The approval number of the local ethics committees for animal experiments of the state of Schleswig-Holstein (Ministerium für Energiewende, Landwirtschaft, Umwelt, Natur und Digitalisierung des Landes Schleswig-Holstein) was 106-10/19.

### 2.2 PCR-based identification of the floxed *C5ar2-tdTomato* gene in sorted immune cells

The PCR-based identification of the floxed *C5ar2-tdTomato* gene in sorted immune cell populations was performed as previously described (50). Briefly, DNA from 1 × 10^5^ sorted immune cells was extracted using the KAPA Express Extract Kit (Peqlab), according to manufacturer’s protocol. To amplify the different DNA fragments, the following primers were used: GK91: 5’-CAAATGTTGCTTGTCTGGTG-3’, GK92: 5’-GTCAGTCGAGTGCACAGTTT-3’, GK360: 5’-TGTCAGCCCGGGACCTTTA-3’, GK361: 5’-CTTATCACGTCCTGCGGGTAA-3’ (Eurofins Scientific). PCRs were run using the following conditions: 95°C for 3 min, followed by 35 cycles at 95°C for 15 sec, 67°C for 15 sec, and 72°C for 10 sec, followed by 72°C for 120 sec. The primer pair GK91/GK92 amplifies a 206-bp DNA fragment of the TCR delta chain gene on chromosome 14 as an internal template control in samples from all strains. The primer pair GK360/GK361 amplifies a 478-bp fragment of the floxed *C5ar2-tdTomato* gene (from exon 2 and the 3’-UTR) including the fragment encoding the *loxP* site. The samples were transferred to a 1.0% sodium borate agarose gel, which was stained for amplification products with GelRed (Biotrend Chemikalien GmbH).

### 2.3 Antibodies

For the antibody transfer model, rabbit anti-murine type VII collagen IgG was used, which was produced and purified as described (51, 52). For flow cytometric analyses, dead cells were stained with the amine-reactive fixable viability dye eFluor 780 (Life Technologies). FcγRs were blocked with anti-mouse CD16/CD32 (5 μg/ml; 93, Life Technologies). Fluorescently labeled antibodies used for flow cytometric analysis or fluorescence-activated cell sorting (FACS) are listed in **Supplementary Table 1**.

### 2.4 Antibody transfer-induced EBA mouse model

WT, *C5ar2-tdTomato^fl/fl^*, and *LysM^cre^-C5ar2^–/–^* mice of either sex were anesthetized and injected subcutaneously with 100 µg affinity-purified rabbit anti-mCOL7 IgG on each days 0, 2, and 4. From day 0, mice were observed every other day for weight, general well-being, and signs of skin lesions (i.e., erythema, blisters, erosions, and crusts), which were assessed as previously described (52). On day 12, tissue biopsies (ear skin, lesions and perilesional skin stored at -80°C) were collected for histopathologic analysis. Immune cells from blood and bone marrow (BM) were isolated for quantitative immunophenotyping by flow cytometry, the latter were also used for *in vitro* stimulation assays.

### 2.5 Histopathology

Ear skin samples were sectioned (6 µm) and differentially stained according to the manufacturer’s protocol of the Kwik-Diff™ Stain Kit (Thermo Scientific, Kalamazoo, MI, USA). Images of the stained tissue sections were acquired and analyzed using the Keyence BZ-X810 all-in-one fluorescence microscope (Keyence, Neu-Isenburg, Germany) and BZ-X800 viewer software after applying white balance to the entire image.

### 2.6 Bone marrow cell preparation

Isolation of mouse BM cells was performed as previously described (53). Briefly, femurs and tibias were rinsed with DPBS containing 2 mM EDTA using a 27G needle. To obtain a single cell suspension and exclude bone fragments, the suspension was filtered through a 40-µm cell strainer. Subsequently, a hypotonic erythrocyte lysis buffer (155 mM NH_4_Cl, 10 mM KHCO_3_, 0.1 mM EDTA at pH 7.2) was used to lyse the contained erythrocytes. For subsequent experiments, the isolated BM cells were stored in DPBS or complete RPMI 1640 medium (RPMI 1640 containing 10% fetal calf serum, 2 mM L-glutamine, 100 U/mL penicillin and 100 µg/mL streptomycin).

### 2.7 Flow cytometry-based immunophenotyping and fluorescence-activated cell sorting

For flow cytometric immunophenotyping and fluorescence-activated cell sorting (FACS) of isolated cells, cells were stained with a fixable viability dye (eF780; Life Technologies) and then incubated with the respective antibodies (see **Supplementary Table 1**) for 15 min at 4°C in the dark. For samples in which Fcγ receptor expression was not of interest, nonspecific binding of antibodies to the Fcγ receptors was prevented by blocking the FcγRs with F_c_-block (unlabeled anti-CD16/CD32 antibody) for 15 min at 4°C. Stained cells were washed and then resuspended in DPBS/1%BSA for flow cytometric analysis on a BD LSRII conventional flow cytometer or a Cytek Aurora spectral analyzer or sorted on a BD AriaIII sorter. Sorting was performed using a 70 µm nozzle.

### 2.8 Assessment of intracellular calcium changes in BM neutrophils

To measure the increase in intracellular calcium (Ca^2+^)_i_ concentration, neutrophils (Ly6G^+^ cells) from isolated BM cells of WT and *LysM^cre^-C5ar2^–/–^* mice were stimulated with recombinant C5a (0.2 nM; Hycult Biotech, Uden, Netherlands), as previously described (53). In brief, BM cells were first stained for neutrophil surface markers, resuspended in DPBS to a final concentration of 1 × 10^7^ cells/mL, and then incubated with 10 µM Fluo-4 AM for 30 minutes at room temperature in the dark. After a wash and repeated incubation step, the fluorescent compound remained in the cytoplasm as intracellular esterases hydrolyzed the acetoxymethyl, exposing the negatively charged carboxylate groups and rendering Fluo-4 cell-impermeant. By measuring the fluorescence signal of Fluo-4 in neutrophils before and after the addition of C5a with a BD LSR II flow cytometer and calculating the relative increase in the AUC of the fluorescence signal with the Kinetics tool in FlowJo software (version 10; Tree Star, Ashland, OR), the increase in (Ca^2+^)_i_ concentration was quantified.

### 2.9 Neutrophil CD11b upregulation assay

Differential expression of the integrin CD11b on neutrophils from WT, *C5ar2-tdTomato^fl/fl^*, and *LysM^cre^-C5ar2^–/–^* mice after stimulation with C5a served as an indicator of cellular activation and was measured by flow cytometry. Briefly, BM cells were incubated in complete RPMI 1640 medium for 30 minutes at 37°C, 5% CO_2_ supplemented with or without 2.5 nM C5a. After washing and blocking FcγRs with anti-CD16/32 antibodies, cells were stained for Ly6G and CD11b. Surface expression of CD11b on neutrophils (Ly6G^+^ cells) was determined using a BD LSRII flow cytometer. Calculation of the relative difference in the geometric mean fluorescence intensity (gMFI) of CD11b expression from stimulated and unstimulated neutrophils then revealed the relative, C5a-induced upregulation of CD11b on neutrophils.

### 2.10 Neutrophil chemotaxis assay

Chemotaxis of neutrophils from WT, *C5ar2-tdTomato^fl/fl^*, and *LysM^cre^-C5ar2^–/–^* mice toward C5a was determined using a transwell assay as previously described (54). In brief, 2 × 10^6^ BM cells were transferred to an insert of a 96-well transwell plate with a pore size of 3 µm (Corning Inc., Kennebunk, ME, USA). The bottom wells of the samples contained 12.5 nM C5a in complete RPMI 1640 medium, whereas the control wells contained only complete RPMI 1640 medium. Cells were incubated at 37°C, 5% CO_2_ for 30 minutes and then collected separately from the transwell insert (non-migrated cells) and the bottom wells (migrated cells). After staining for Ly6G and CD11b, neutrophils were quantified using a Cytek Aurora spectral flow cytometer. To obtain the relative amount of chemotactic neutrophils, the number of migrated neutrophils was divided by the total number of neutrophils recovered (non-migrated and migrated). In addition, the values obtained were corrected for the number of neutrophils that migrated through the pores passively due to chemokinesis.

### 2.11 Statistical analysis

GraphPad Prism software (version 9.5.1; GraphPad Software, San Diego, CA, USA, www.graphpad.com) was used for statistical analysis of the data. All plots show mean values ± standard error of the mean (SEM). Data sets were analyzed for outliers using the ROUT method with a false discovery rate (FDR) of Q = 5%. Outliers were excluded from the analysis. The unpaired Mann-Whitney test was used to determine the *p*-values of comparisons between two independent groups. For comparisons between more than two independent groups, the Kruskal-Wallis test with Dunn’s multiple comparison test was used, and for comparisons involving different time points or different subpopulations, the two-way ANOVA with Holm-Šídák multiple comparison test was used. Significance was assumed when the *p*-value was < 0.05 (* *p* < 0.05, ** *p* < 0.01, *** *p* < 0.001, **** *p* < 0.0001).

## 3 Results

### 3.1 Generation and characterization of a neutrophil-specific *C5aR2* knock-out mouse line

**Figure 1:**
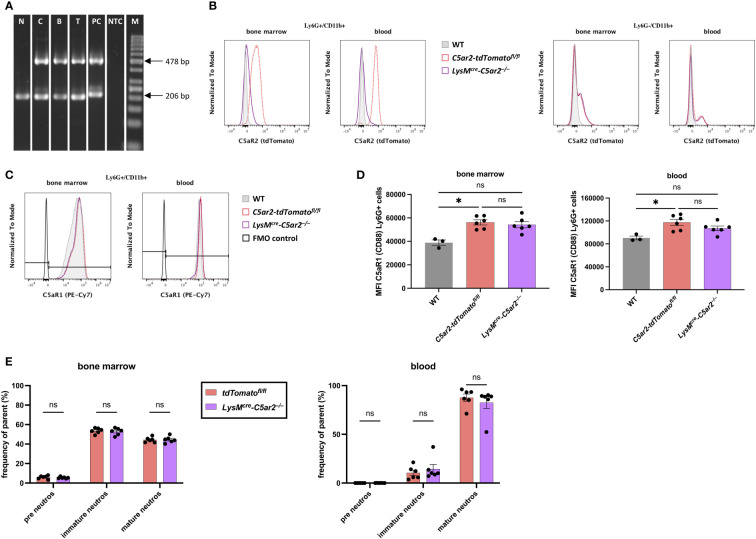
Characterization of the cell-specific C5aR2 knock-out mouse line. **(A)** PCR-based detection of the floxed *tdTomato-C5ar2* gene cassette in BM neutrophils (N), BM Ly6G^-^/CD11b^+^ cells (C), splenic B (B) and T cells (T) from *LysM* ^cre^ *-C5ar2* ^–/–^ mice. Positive control from *C5ar2-tdTomato* ^fl/fl^ mice (PC) and No Template Control (NTC) are shown. M: GeneRuler 50 bp DNA Ladder. **(B)** Histogram overlay of tdTomato reporter signal indicating C5aR2 expression in BM and blood neutrophils (Ly6G^+^/CD11b^+^) and Ly6G^-^/CD11b^+^ cells from *LysM* ^cre^ *-C5ar2* ^–/–^ (purple) and *C5ar2-tdTomato* ^fl/fl^ (red) mice. The tdTomato signal of corresponding cells from WT (grey) mice served as a negative control. **(C)** Histogram overlay indicating C5aR1 expression in BM and blood neutrophils from *LysM* ^cre^ *-C5ar2* ^–/–^ (purple), *C5ar2-tdTomato* ^fl/fl^ (red), and WT (grey) mice. The signal corresponding to the FMO control is shown in black. **(D)** C5aR1 expression in BM and blood neutrophils (Ly6G^+^ cells) from *LysM* ^cre^ *-C5ar2* ^–/–^, *C5ar2-tdTomato* ^fl/fl^, and WT mice quantified by the gMFI. **(E)** Frequency (of parent) of neutrophil subsets in the BM and blood of naïve *LysM* ^cre^ *-C5ar2* ^–/–^ and *C5ar2-tdTomato* ^fl/fl^ control mice (n = 6/group). * p < 0.05; ns, not significant.

We generated a LysM-specific *C5aR2* knock-out mouse line by *in vitro* fertilization of female *C5ar2-tdTomato^fl/fl^* mice with sperm of *LysM^cre^* mice and subsequent breeding of a homozygous *LysM^cre^-C5ar2^–/–^* mouse line. In *C5ar2-tdTomato^fl/fl^* mice a sequence encoding the reporter protein tandem dimer Tomato (tdTomato) has been inserted in-frame with the coding sequence of *C5ar2* immediately following the splice acceptor of exon 2 (4). Because the coding sequences of *tdTomato* and *C5ar2* are separated by a sequence encoding the self-cleaving peptide from porcine teschovirus-1 (P2A), both the reporter protein tdTomato and C5aR2 are expressed as individual protein products in this mouse once the corresponding mRNA is transcribed (4). In addition, the entire gene cassette is floxed with loxP sites, allowing specific knockout of the entire locus by cell-specific expression of Cre recombinase (55). We sorted BM neutrophils (Ly6G^+^, CD11b^+^), BM CD11b^+^ cells (Ly6G^-^, CD11b^+^), splenic B cells (CD19^+^, CD45R^+^) and splenic T cells (CD3^+^; **Supplementary Figures 1A, B**) and analyzed the cell-specific deletion of the *C5ar2* gene by genotyping. We found a signal corresponding to the floxed *C5ar2-tdTomato^fl/fl^* gene construct (478 bp) in splenic B and T cells as well as in BM Ly6G^-^/CD11b^+^ cells but could not detect this sequence in BM neutrophils (**Figure 1A**).

We also checked for C5aR2 expression in WT, *C5ar2-tdTomato^fl/fl^*, and *LysM^cre^-C5ar2^–/–^* mice by analyzing the surrogate marker tdTomato signal in BM and blood neutrophils (Ly6G^+^, CD11b^+^) by flow cytometry (**Figure 1B**). We found a clear tdTomato signal in BM (approximately 75% tdTomato^+^ neutrophils) and particularly in blood neutrophils (>95% tdTomato^+^ neutrophils) from *C5ar2-tdTomato^fl/fl^* mice, whereas the tdTomato signal in neutrophils from *LysM^cre^-C5ar2^–/–^* mice corresponded to the negative tdTomato signal in neutrophils from WT animals. In Ly6G^-^, CD11b^+^ cells, we did not detect differences between *LysM^cre^-C5ar2^–/–^* and *C5ar2-tdTomato^fl/fl^* control mice, with only a subfraction of cells staining positive for tdTomato in each case. Overall, this confirms the cell-specific deletion of the *C5ar2* gene also at the protein level.

Phenotypically, the newly created *LysM^cre^-C5ar2^–/–^* mice appeared healthy and were not different from either *C5ar2-tdTomato^fl/fl^* or WT mice. However, as both the *C5ar1* and *C5ar2* gene loci are located on chromosome 7 in close proximity (only about 15 kb apart), we additionally compared the C5aR1 expression pattern in WT; *C5ar2-tdTomato^fl/fl^* and *LysM^cre^-C5ar2^–/–^* mice. In both BM and blood neutrophils, >99% of cells stained positive for C5aR1. At steady state, surface expression of C5aR1 was comparable high in BM and blood neutrophils (Ly6G^+^ cells) from *LysM^cre^-C5ar2^–/–^* mice compared with respective cells from *C5ar2-tdTomato^fl/fl^* and WT mice (**Figures 1C, D**). Of note, we detected a significant difference in surface expression of C5aR1 between neutrophils from *C5ar2-tdTomato^fl/fl^* and WT mice based on the gMFI. We additionally checked whether neutrophil development in the BM niche may be influenced by Cre recombinase activity under the control of the *LysM* promoter and cell-specific deletion of *C5ar2* in neutrophils. Based on the percentage of detected neutrophil precursors (pre neutros), immature neutrophils (immature neutros), and mature neutrophils (mature neutros), no differences in neutrophil development were detected (**Figure 1E**).

### 3.2 *C5aR2* deficiency in neutrophils ameliorates pathogenesis in an experimental model of EBA

**Figure 2:**
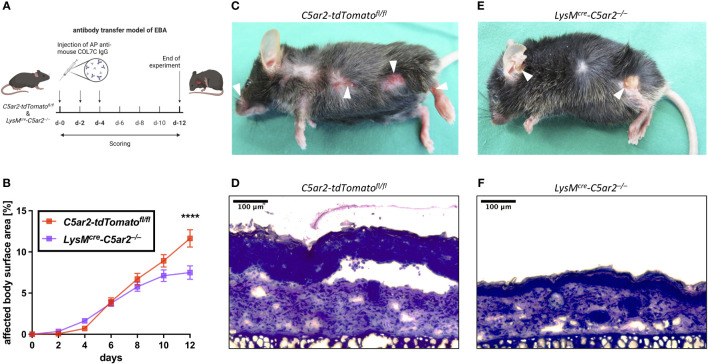
*C5aR2* deficiency in neutrophils ameliorates pathogenesis in an experimental model of EBA. **(A)** Schematic representation of the antibody transfer mouse model of EBA. Subepidermal blistering is induced by subcutaneous injection of affinity-purified rabbit anti-mCOL7 IgG on days (d) 0, 2, and 4. Cutaneous lesions are scored every other day. Mice are sacrificed on d-12 (figure created with BioRender.com). **(B)** Development of the clinical disease phenotype as indicated by the percentage of body surface area affected by lesions, erosions, and blisters in *LysM^cre^-C5ar2^–/–^* and *C5ar2-tdTomato^fl/fl^* control mice (n = 15 per group). Representative pictures of clinical lesions found in **(C)** *C5ar2-tdTomato^fl/fl^* and **(E)** *LysM^cre^-C5ar2^–/–^* mice. Example photographs of histopathologically examined skin sections from **(D)** *C5ar2-tdTomato^fl/fl^* and **(F)** *LysM^cre^-C5ar2^–/–^* mice. **** p < 0.0001.

We used the established *LysM^cre^-C5ar2^–/–^* mouse line in the antibody transfer model of EBA (**Figure 2A**) to confirm our previous hypothesis that the lack of *C5ar2* in neutrophils is responsible for the decreased disease severity reported in mice with a global *C5aR2* deficiency. In these experiments *C5ar2-tdTomato^fl/fl^* mice served as a control.

Signs of skin blistering (erosion, lesions, and/or crusts) were detectable as early as 2 days post anti-mCOL7 IgG injection in mice of both genotypes. Of note, similar to mice with global *C5aR2* deficiency (35), *LysM^cre^-C5ar2^–/–^* mice showed an overall ameliorated disease progression (**Figure 2B**). Interestingly, however, and in contrast to *C5ar2^–/–^* mice disease onset was similar in *LysM^cre^-C5ar2^–/–^* mice when compared to *C5ar2-tdTomato^fl/fl^* mice and was slowed only after day 6. By day 12, significantly less areas of blister-affected skin (7.5 ± 0.8%) were detected in *LysM^cre^-C5ar2^–/–^* mice compared to *C5ar2-tdTomato^fl/fl^* mice (11.6 ± 1.0%), which showed a similar disease progression as we previously reported for WT mice (35).

The significant difference in the affected body surface area found on day 12 is exemplified by representative photographs of mice from both genotypes showing that the distribution of skin lesions on the body surface was comparable, but numbers and severity of blisters were clearly higher in *C5ar2-tdTomato^fl/fl^* control mice (**Figures 2C, E**). In addition, histological analysis of skin sections of the ear showed evident detachment of the epidermis from the dermis at the dermal-epidermal junction (DEJ), swelling of the tissue, and cellular infiltration in samples from *C5ar2-tdTomato^fl/fl^* control mice but not in samples from *LysM^cre^-C5ar2^–/–^* mice, suggesting ameliorated disease in the latter (**Figures 2D, F**). Taken together, these data demonstrate that the deficiency of *C5aR2* in neutrophils leads to an ameliorated disease phenotype in the antibody transfer model of EBA when compared with *C5ar2-tdTomato^fl/fl^* control mice.

### 3.3 *In vitro* chemotaxis of neutrophils from *LysM^cre^-C5ar2^–/–^* mice toward C5a is unchanged

**Figure 3:**
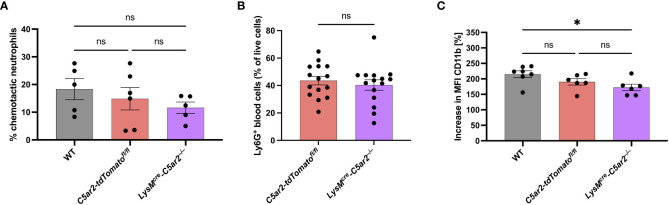
C5a-mediated *in vitro* activation of neutrophils from *LysM^cre^-C5ar2^–/–^* mice. **(A)** *In vitro* chemotaxis of BM cells of *LysM^cre^-C5ar2^–/–^*, *C5ar2-tdTomato^fl/fl^* and WT control mice towards C5a (n ≥ 5/group). **(B)** Neutrophil frequency in the blood of diseased (EBA) *LysM^cre^-C5ar2^–/–^* and *C5ar2-tdTomato^fl/fl^* control mice (n = 15/group). **(C)** C5a-induced increase in CD11b surface expression of *LysM^cre^-C5ar2^–/–^*, *C5ar2-tdTomato^fl/fl^* and WT control mice (n ≥ 6/group). * p < 0.05; ns, not significant.

Because we previously observed reduced C5a-mediated chemotaxis of BM neutrophils from *C5ar2^–/–^* mice compared to neutrophils from WT mice *in vitro*, we aimed to verify this by employing BM neutrophils from *LysM^cre^-C5ar2^–/–^* mice to the same chemotaxis assay. Here, we did not detect a significant difference in chemotaxis toward C5a between BM neutrophils from *LysM^cre^-C5ar2^–/–^* and *C5ar2-tdTomato^fl/fl^* or WT control mice, although there was a non-significant trend toward lower chemotaxis of neutrophils from *LysM^cre^-C5ar2^–/–^* mice (**Figure 3A**).

In addition to the *in vitro* chemotaxis assay we also checked for neutrophil numbers in the blood of diseased *LysM^cre^-C5ar2^–/–^* and *C5ar2-tdTomato^fl/fl^* control mice. We did not detect any differences in the number of neutrophils found in the blood being in line with our previous report (35; **Figure 3B**).

### 3.4 C5aR2 deficiency on neutrophils reduces C5a-mediated cellular activation

We reported previously that BM neutrophils from *C5ar2^–/–^* mice exhibit reduced cellular activation following stimulation with C5a (and C5a^desArg^; 15), which prompted us to hypothesize that this is partly the reason for the ameliorated disease phenotype observed in the EBA mouse model with these mice (35). In line with this hypothesis, we here found a significantly ameliorated disease phenotype for *LysM^cre^-C5ar2^–/–^* mice. However, as C5a-induced chemotaxis of neutrophils was not affected by the cell-specific deletion of *C5ar2* in these mice, we tested for the cellular activation of BM neutrophils from *LysM^cre^-C5ar2^–/–^* mice after C5a stimulation. C5a-induced up-regulation of CD11b surface expression was significantly diminished in BM neutrophils from *LysM^cre^-C5ar2^–/–^* mice compared with WT but not *C5ar2-tdTomato^fl/fl^* control mice (**Figure 3C**). The mean up-regulation of CD11b surface expression was 172.3 ± 9.7% in neutrophils from *LysM^cre^-C5ar2^–/–^* mice, whereas surface expression of CD11b increased by 190.3 ± 9.5% and 215.1 ± 10.0% in neutrophils from *C5ar2-tdTomato^fl/fl^* and WT control mice, respectively. We also checked for C5a-induced calcium flux in neutrophils from *LysM^cre^-C5ar2^–/–^* and WT mice. The mean increase in the calcium flux, as measured by the calcium-sensitive dye Fluo-4 AM, was 85.9 ± 6.2% in neutrophils from *LysM^cre^-C5ar2^–/–^* mice, while neutrophils from WT control mice showed a mean increase of 162.8 ± 11.8% after C5a stimulation (**Supplementary Figure S2**).

### 3.5 Fcγ receptor expression is altered in neutrophils with C5aR2 deficiency

**Figure 4:**
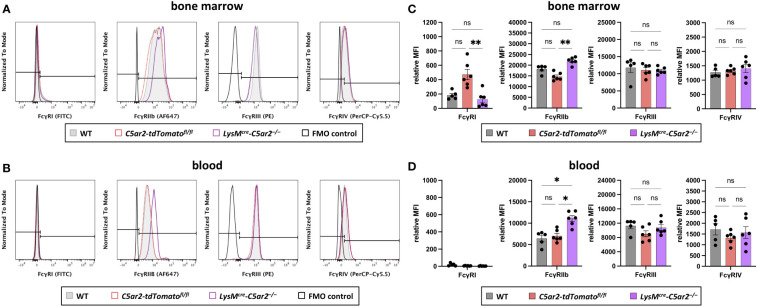
Fcγ receptor expression on BM and blood neutrophils. Histogram overlays of surface expression of FcγRs (FcγRI, FcγRIIb, FcγRIII, FcγRIV) on neutrophils from **(A)** BM and **(B)** blood of WT, *C5ar2-tdTomato^fl/fl^*, and *LysM^cre^-C5ar2^–/–^* mice. The signal corresponding to the FMO control is shown in black. Relative gMFI of FcγRs corresponding to surface expression on **(C)** BM and **(D)** blood neutrophils of WT, *C5ar2-tdTomato^fl/fl^*, and *LysM^cre^-C5ar2^–/–^* mice. * p < 0.05, ** p < 0.01; ns, not significant.

Recently, we published that neutrophils isolated from *C5ar2^–/–^* mice exhibit a changed expression pattern in Fcγ receptors, resulting in a decreased release of ROS – critical drivers of tissue destruction in EBA pathogenesis (47, 56) – after stimulation with ICs consisting of murine COL7C and rabbit anti-mouse COL7 IgGs (35). Using *LysM^cre^-C5ar2^–/–^* mice we tested if the change in FcγR expression is a direct effect resulting from the deficiency in *C5ar2* in neutrophils or whether this is a consequence of global deficiency in *C5ar2*. Only 10%, 27%, and 15% of BM neutrophils from *LysM^cre^-C5ar2^–/–^*, *C5ar2-tdTomato^fl/fl^*, and WT mice, respectively, stained positive for FcγRI **Figure 4A**. Surface expression of FcγRI in these cells quantified by the relative gMFI was low. However, there was a significant difference in the relative gMFI for FcγRI between cells from *LysM^cre^-C5ar2^–/–^* and *C5ar2-tdTomato^fl/fl^* control mice, with cells from *C5ar2-tdTomato^fl/fl^* control mice showing an increased relative gMFI. Almost all blood neutrophils (98.5-99.5%) were negative for FcγRI, regardless of the genotype from which these cells were isolated (**Figure 4B**). FcγRIIb and FcγRIII were expressed on >99.5% and >98.5%, respectively, of all BM neutrophils from mice of all three genotypes. Interestingly, based on the relative gMFI, surface expression of inhibitory FcγRIIb was significantly increased in BM neutrophils from *LysM^cre^-C5ar2^–/–^* mice compared with neutrophils isolated from *C5ar2-tdTomato^fl/fl^* or WT mice (**Figures 4C**). Surface expression of FcγRIIb was lowest in neutrophils from *C5ar2-tdTomato^fl/fl^* mice. Although the surface expression of FcγRIIb on neutrophils decreased once neutrophils transitioned from the BM to the blood, ≥99.5% of cells still expressed FcγRIIb, and the effect of significantly increased expression on the surface of neutrophils from *LysM^cre^-C5ar2^–/–^* mice persisted (**Figure 4D**). FcγRIII expression on blood neutrophils was similar to BM neutrophils. Surface expression of FcγRIV was found on the majority of BM and blood neutrophils, regardless of the genotype from which the cells were isolated. Based on the relative gMFI, FcγRIV surface expression on blood neutrophils was increased compared with their BM counterparts. However, no difference in surface expression of FcγRIV was detected between *LysM^cre^-C5ar2^–/–^*, *C5ar2-tdTomato^fl/fl^*, and WT mice (**Figure 4A**).

Taken together, these data confirm that deficiency of *C5ar2* on neutrophils alters the expression level of Fcγ receptors, leading in particular to increased surface expression of inhibitory FcγRIIb.

## 4 Discussion

We recently reported that *C5ar2^–/–^* mice have an ameliorated disease phenotype in the antibody-transfer model of EBA. Based on the critical role of neutrophils in EBA pathogenesis (47, 56–58) and the results of our *in vitro* data analyzing the C5a and Fcγ receptor-mediated activation of neutrophils, we hypothesized that a deficiency of *C5ar2* in neutrophils is the leading cause for the ameliorated disease phenotype. Here, we confirmed our previous hypothesis by generating a mouse line, *LysM^cre^-C5ar2^–/–^*, in which *C5ar2* is specifically deleted in *LysM*-expressing cells. Like mice with a global deficiency in *C5ar2*, mice of this newly generated mouse line showed an ameliorated disease phenotype in the antibody transfer mouse model of EBA. However, a limitation of using the *LysM^cre^* mice is that the *LysM*/*Lyz2* promoter is expressed in cells of the myeloid lineage, including not only neutrophils but also monocytes/macrophages (59, 60). And although we still detected the tdTomato coding sequence in Ly6G^-^/CD11b^+^ BM cells (**Figure 1B**), it is still reasonable to assume that some of the observations made with these mice using the antibody transfer mouse model of EBA are due to altered monocyte/macrophage function (34). Yet, there is limited evidence for a functional role of monocyte-derived cells in the pathogenesis of EBA (61, 62). Comparison of the use of anti-Ly6G (1A8) and anti-Ly6C/G (RB6-8C5) depleting antibodies suggests that monocyte depletion has an additional beneficial effect in experimental EBA (47, 58, 62). Moreover, monocytes have been shown to induce subepidermal blisters in an *ex vivo* model of EBA (61). Therefore, it cannot be completely excluded that monocytes or monocyte-derived cells that have been shown to express *C5ar2* (4, 63) also contribute in part to the attenuated disease phenotype observed in global and cell-specific *C5ar2* knockout mice, although the main drivers of the disease are clearly neutrophils (34). In addition, we used *C5ar2-tdTomato^fl/fl^* mice but not *LysM^cre^* mice as controls. In *LysM^cre^* mice, the function of the endogenous *LysM* gene (chromosome 10) is abolished (59, 64). Although an effect of lysozyme, a glycoside hydrolase that catalyzes the hydrolysis of the β-1,4-glycosidic bond between N-acetylmuramic acid and N-acetylglucosamine, on the pathogenesis of EBA seems unlikely, and *LysM^cre^* mice showed a similar response to WT animals in an LPS-induced model of sterile acute lung injury (64), an influence of the *LysM^cre^* allele on disease progression in the EBA model cannot be completely excluded.

Remarkably, the differences in disease progression between WT and *LysM^cre^-C5ar2^–/–^* mice began after an initiation phase that was similar in both mouse strains. Because this effect was also found when comparing WT and *C5ar2^–/–^* mice (35), we hypothesize that this is an effect caused by the sustainment of neutrophil recruitment and activation, possibly controlled by CD11b and FcγRs. In turn, the expression of CD11b and FcγRs appears to be influenced by C5a receptor expression and/or signaling. Consistent with this and our results in the chemotaxis assay, Chen et al. found no difference in neutrophil recruitment to the peritoneum between WT and *C5ar2^–/–^* mice after *i.p.* injection of C5a. However, using an air-pouch model, they showed that after injection of C5a alone or together with thioglycolate into the air-pouches, sustained neutrophil recruitment was significantly reduced in *C5ar2^–/–^* mice compared with WT mice (30). This suggests that although C5aR2 deficiency does not affect short-term neutrophil recruitment, it significantly reduces long-term sustained neutrophil recruitment, which may influence disease progression in the antibody transfer model of EBA.

In our previous publication, we reported reduced cellular activation in BM neutrophils from *C5ar2^–/–^* mice that was dependent on the presence of C5aR1 (35). We have shown that C5a-induced up-regulation of CD11b is reduced in neutrophils from *C5ar2^–/–^* mice compared with neutrophils from WT animals and is completely absent in neutrophils from *C5ar1^–/–^* mice. In addition, we found an altered expression pattern of FcγRs, favoring a more anti-inflammatory phenotype in neutrophils from *C5ar2^–/–^* mice. Here, we confirm our previous findings by using mice with LysM-specific *C5ar2* deficiency. Neutrophils from these mice also exhibited reduced C5a-induced CD11b up-regulation and an altered expression pattern of FcγRs characterized by increased expression of the inhibitory Fcγ receptor FcγRIIb. This was an interesting finding as it links the function of C5aR2 to both Mac-1 (CD11b/CD18) and FcγRs.

In experimental EBA, the activating Fcγ receptors including FcγRIII and FcγRIV promote disease development, whereas the inhibitory FcγRIIb has been shown to convey some level of protection (65, 66). A bi-directional crosstalk between C5a and Fcγ receptors has been described earlier, suggesting that IC-induced C5a tips the balance between activating and inhibitory FcγRs (A/I ratio) toward the activating phenotype (40, 67–70). This results in a self-amplifying feedback loop sustaining C5a generation and priming the cells for an inflammatory response (69–72). Moreover, in addition to neutrophil activation it has been proposed that the crosstalk between C5a and Fcγ receptors also sustains neutrophil recruitment *in vivo*, which relied on C5a-induced secretion of leukotriene B4 (LTB_4_) by neutrophils (73).

Intriguingly, the C5a/C5aR1-axis has previously been shown to play an important role in the transition of neutrophils from firm arrest to spreading and crawling to enable subsequent extravasation in a LTB_4_/BLT1-dependent manner (74). Importantly, this process was dependent on the crosstalk with Mac-1 and LFA-1, indicating that both β_2_-integrins are activated by C5a-stimulation in neutrophils and that this is an important regulatory step in C5a-triggered diapedesis into inflamed tissue (74).

In addition, Mac-1 has also been described to interact with several other immune receptors on neutrophils, including Fcγ receptors, CD14, Dectin-1, and TLRs, which might explain why Mac-1 is not only involved in neutrophil migration but also affects important effector functions of neutrophils (42). Considering IC-mediated autoimmune diseases such as EBA, crosstalk with Fcγ receptors is of particular interest, as it has been shown that the activating Fcγ receptors in mice are critical for neutrophil accumulation in models of IC-induced inflammation using mice lacking the common γ-chain (42). It has been shown in the past that Mac-1 is not essential for the initial FcγR-mediated binding to ICs but is required for sustained firm interaction with and activation of neutrophils by these ICs, which likely involves paxilin-induced cytoskeleton remodeling (45, 48, 75–78). Moreover, in an antibody transfer model of acute anti-glomerular basement membrane glomerulonephritis, the sustainment of neutrophil interaction with deposited ICs was abolished in the absence of Mac-1 (45, 77) and prevented the local release of ROS associated with renal damage in this model (42, 77). Moreover, blockade of CD18 has been shown not to affect ROS production or neutrophil elastase release from IC-activated neutrophils *in vitro* (48). However, IC-induced neutrophil adhesion, which limits protease inhibitor access and is critical for anti-COL7 IgG-mediated tissue injury in an *ex vivo* model, was significantly affected by blockade of CD18 *in vitro* and *in vivo* (48). Accordingly, we hypothesize that the altered expression pattern of FcγRs and the lower C5a-induced expression of CD11b on neutrophils from *LysM^cre^-C5ar2^–/–^* mice affects the IC-mediated neutrophil adhesion and, consequently, the duration rather than the magnitude of local neutrophil activation in tissues, particularly contributing to the late phase of disease progression.

Based on these previous findings and our results, we propose an important interaction network between ICs, FcγRs, C5a receptors, and β_2_-integrins (including Mac-1 and potentially also LFA-1) that appears to be critical for disease development in experimental EBA. In this model, deposition of ICs in the skin leads to local activation of complement, in particular C3b and its degradation products, which deposit along the DEJ, and C5a, which causes mobilization and activation (up-regulation of CD11b, increase in the A/I-ratio) of neutrophils. Activated neutrophils migrate to the site of inflammation, a process promoted by β_2_-integrins and FcγRs and supported by chemokines such as LTB_4_. In the skin, neutrophils accumulate at the DEJ, where sustained interaction with and activation by deposited ICs and C3b is achieved by FcγR/Mac-1 interaction, resulting in local adhesion and the continuous release of ROS and proteases that destroy the tissue in an enclosed space protected from ubiquitously expressed proteinase inhibitors (48). In tissue, activated neutrophils also release IL-1β, which stimulates fibroblasts to release cytokines that contribute to the sustainment of neutrophil influx and inflammation (74). Accordingly, anything that disturbs this interaction network also has a critical impact on disease development: IC deposition at the DEJ is a prerequisite for complement activation and neutrophil recruitment to trigger subepidermal blister formation (38, 79, 80); deficiency of the activating Fcγ receptor FcγRIV (and FcγRIII) protects from disease development, whereas deficiency of FcγRIIb significantly exacerbates disease (65, 66); deficiency of CD18 abrogates infiltration of neutrophils into the skin and local adhesion to deposited ICs, thus preventing disease development (47, 48); deficiency in C5aR1 protects from disease development (40), whereas deficiency in C5aR2 significantly ameliorates pathogenesis of EBA, despite similar blood neutrophilia and similar amounts of ICs deposited at the DEJ (35).

Consistent with this view, neutrophils from *C5ar1^–/–^* mice show neither C5a-induced cellular activation (intracellular calcium flux, up-regulation of CD11b) nor directional chemotaxis toward C5a *in vitro* (35). Moreover, we showed that neutrophils from mice with a global (*C5ar2^–/–^*) or neutrophil-specific (*LysM^cre^-C5ar2^–/–^*) deficiency of C5aR2 exhibit a partial inhibition of C5a-induced up-regulation of CD11b, an A/I-ratio of FcγRs shifted to a more inhibitory phenotype, and reduced chemotaxis toward C5a and C5a^desArg^ (15, 35). Thus, signaling through C5a receptors induces a local inflammatory milieu, primes neutrophils for an inflammatory response, and facilitates directional chemotaxis within the tissue. All of this appears to be primarily dependent on the availability of C5aR1, whose function is promoted by C5aR2 in these settings (34).

In summary, the findings presented here confirm our previous hypothesis that C5aR2 plays a critical role in regulating neutrophil activation and function that contributes to neutrophil-driven autoimmune diseases such as EBA. However, although we have shown that *C5aR2* expression in LysM-positive cells appears to be determinant of disease progression, and mechanistically this appears to be related to the C5a-dependent control of CD11b and FcγRIIb expression, further experiments are needed to fully understand the still enigmatic role of C5aR2 in the regulation of the neutrophil-driven immune response.

## Data availability statement

The raw data supporting the conclusions of this article will be made available by the authors, without undue reservation.

## Ethics statement

The animal study was reviewed and approved by Ministerium für Energiewende, Landwirtschaft, Umwelt, Natur und Digitalisierung des Landes Schleswig-Holstein.

## Author contributions

Conceptualization: CK, DS, JK. Funding Acquisition: CK. Investigation: DS, KK, MK. Project Administration: DS, CK. Resources: RL, KB, JK. Supervision: DL, CK. Writing – Original Draft Preparation: DS. Writing – Review and Editing: DS, KK, MK, CS, RL, JK, CK. All authors contributed to the article and approved the submitted version.
