# Supplementary figures and images for: The complement receptor C5aR2 regulates neutrophil activation and function contributing to neutrophil-driven epidermolysis bullosa acquisita
Source: Front Immunol. 2023 May 19;14:1197709. doi: 10.3389/fimmu.2023.1197709 (PMC10235453; doi:10.3389/fimmu.2023.1197709)

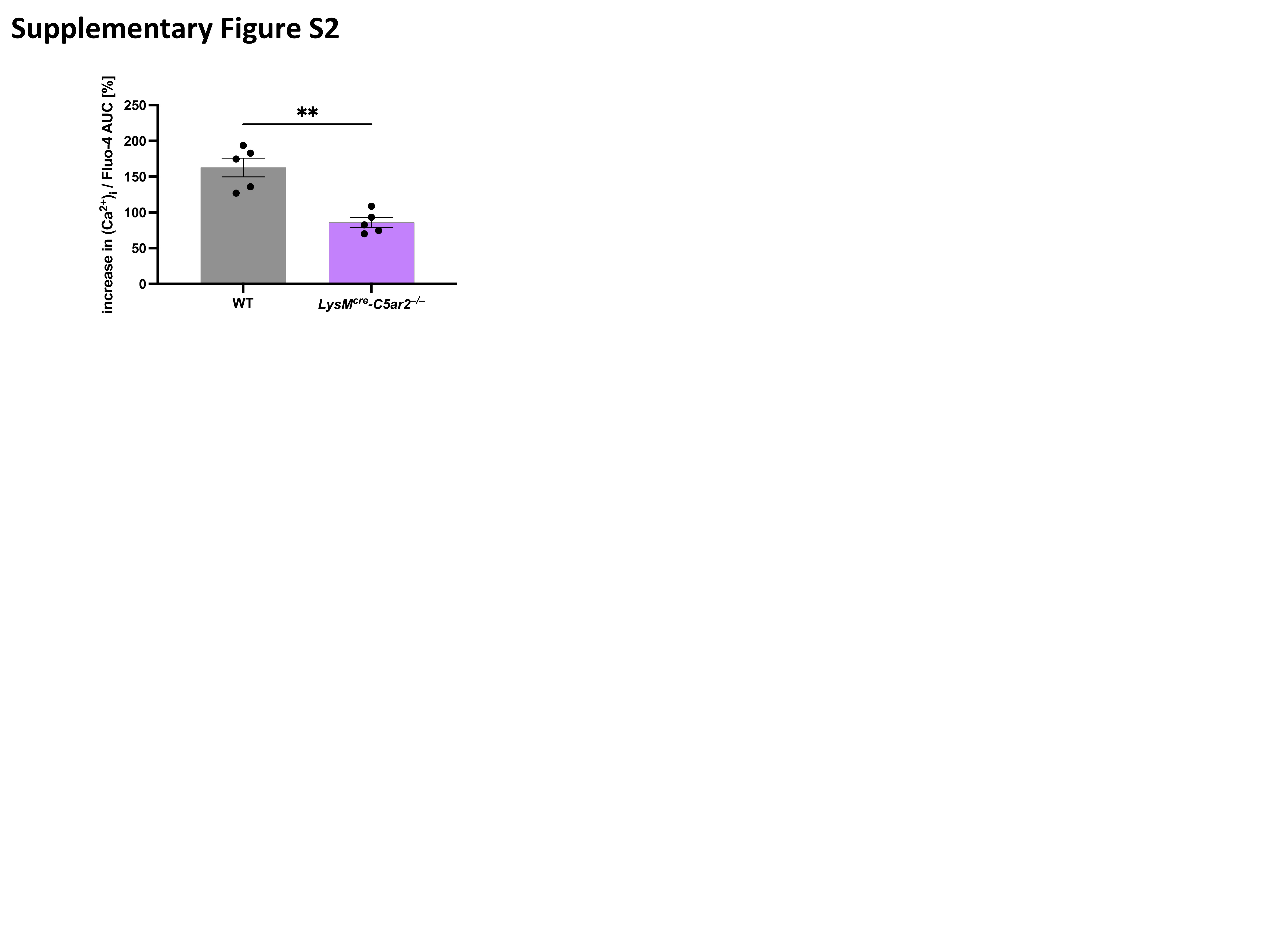

Supplement: Supplementary file 2 [file Image_2.jpg]
