# Supplementary material for: The complement receptor C5aR2 regulates neutrophil activation and function contributing to neutrophil-driven epidermolysis bullosa acquisita
Source: Front Immunol. 2023 May 19;14:1197709. doi: 10.3389/fimmu.2023.1197709 (PMC10235453; doi:10.3389/fimmu.2023.1197709)
Supplement: Supplementary file 3 [file Table_1.docx]

Supplementary Material

The complement receptor C5aR2 regulates neutrophil activation and function contributing to neutrophil-driven autoimmunity

Daniel L. Seiler^1^, Katja H. Kähler^1^, Marie Kleingarn^1^, Christian D. Sadik^2,3^, Katja Bieber^2,3,4^, Ralf J. Ludwig^2,3,4^, Jörg Köhl^1,5^, Christian M. Karsten^1,*^

^1^Institute for Systemic Inflammation Research (ISEF), University of Lübeck, Lübeck, Germany

^2^Center for Research on Inflammation of the Skin (CRIS), University of Lübeck, Lübeck, Germany

^3^Department of Dermatology, University of Lübeck, Lübeck, Germany

^4^Lübeck Institute of Experimental Dermatology (LIED), University of Lübeck, Lübeck, Germany

^5^Division of Immunobiology, Cincinnati Children’s Hospital Medical Centre, University of Cincinnati College of Medicine, Cincinnati, Ohio, USA

*** Correspondence:**Christian M. Karsten
[christian.karsten@uksh.de](mailto:christian.karsten@uksh.de)

# Supplementary Figures and Tables

## Supplementary Figures

Supplementary Figure S1

Supplementary Figure S1: Analysis of neutrophil development. (a) Gating strategy to identify and sort neutrophils (Ly6G^+^/CD11b^+^) and Ly6G^-^/CD11b^+^ cells in BM as well as B and T cells in the spleen via FACS. (b) Gating strategy to identify neutrophil subsets in the BM and blood of *LysM^cre^-C5ar2^–/–^* and *C5ar2-tdTomato^fl/fl^* control mice according to Evrard et al., 2018.

Supplementary Figure S2

Supplementary Figure S2: C5a-initiated calcium flux in BM neutrophils. (a) Gating strategy to identify neutrophils based on the expression of Ly6G.

## Supplementary Tables

Supplementary Table 1: Fluorochrome-labeled antibodies used for flow cytometric analysis and FACS.

| **Antibody** | **Fluorophore** | **Clone** | **Manufacturer** | **Final Concentration** |
| --- | --- | --- | --- | --- |
| CD3 | eF450 | 145-2C11 | eBioscience | 1 µg/mL |
| CD11b | BV510 | M1/70 | BioLegend | 1 µg/mL |
| CD16.2 (FcγRIV) | PerCP-Cy5.5 | 9E9 | BioLegend | 1 µg/mL |
| CD19 | BV711 | 6D5 | BioLegend | 1 µg/mL |
| CD45R (B220) | APC | RA3-6B2 | eBioscience | 1 µg/mL |
| CD45R (B220) | AF700 | RA3-6B2 | eBioscience | 1 µg/mL |
| CD64 (FcγRI) | FITC | X54-5/7.1 | BioLegend | 2.5 µg/mL |
| CD88 (C5aR1) | PE-Cy7 | 20/70 | BioLegend | 1 µg/mL |
| CD90 | AF700 | 30-H12 | BioLegend | 2.5 µg/mL |
| CD115 | BV605 | AFS98 | BioLegend | 1 µg/mL |
| CD115 | BV711 | AFS98 | BioLegend | 1 µg/mL |
| CD117 | PE-Cy7 | 2B8 | BioLegend | 1 µg/mL |
| CD182 (CXCR2) | PE-Dazzle 594 | SA044G4 | BioLegend | 1 µg/mL |
| CD184 (CXCR4) | BV421 | L276F12 | BioLegend | 1 µg/mL |
| FcγRIIb | AF647 | Ly17.2 | Kindly provided by Falk Nimmerjahn | 2.5 µg/mL |
| Ly6C | BV605 | HK1.4 | BioLegend | 0.25 µg/mL |
| Ly6G | V450 | 1A8 | BD Bioscience | 1 µg/mL |
| Ly6G | APC | 1A8 | BD Bioscience BioLegend | 1 µg/mL |
| NK1.1 | AF700 | PK136 | BioLegend | 2.5 µg/mL |
| SiglecF | BV650 | E50-2440 | BD Bioscience | 1 µg/mL |
